# Identification and functional analysis of genetic variants in TBX5 gene promoter in patients with acute myocardial infarction

**DOI:** 10.1186/s12872-019-1237-6

**Published:** 2019-11-27

**Authors:** Shuai Wang, Jie Zhang, Xiaohui He, Yexin Zhang, Jing Chen, Qiang Su, Shuchao Pang, Shufang Zhang, Yinghua Cui, Bo Yan

**Affiliations:** 1 Department of Medicine, Shandong University School of Medicine, Jinan, 250012 Shandong China; 2 Shandong Provincial Key Laboratory of Cardiac Disease Diagnosis and Treatment, Affiliated Hospital of Jining Medical University, Jining Medical University, 89 Guhuai Road, Jining, 272029 Shandong China; 3 Shandong Provincial Sino-US Cooperation Research Center for Translational Medicine, Affiliated Hospital of Jining Medical University, Jining Medical University, Jining, 272029 Shandong China; 4 Division of Cardiology, Affiliated Hospital of Jining Medical University, Jining Medical University, 89 Guhuai Road, Jining, 272029 Shandong China; 5 Center for Molecular Genetics of Cardiovascular Diseases, Affiliated Hospital of Jining Medical University, Jining Medical University, Jining, 272029 Shandong China

**Keywords:** Acute myocardial infarction, TBX5, Gene expression regulation, Promoter, Genetic variants

## Abstract

**Background:**

Coronary artery disease (CAD), including acute myocardial infarction (AMI), is a common complex disease. Although a great number of genetic loci and variants for CAD have been identified, genetic causes and underlying mechanisms remain largely unclear. Epidemiological studies have revealed that CAD incidence is strikingly higher in patients with congenital heart disease than that in normal population. T-box transcription factors play critical roles in embryonic development. In particular, TBX5 as a dosage-sensitive regulator is required for cardiac development and function. Thus, dysregulated TBX5 gene expression may be involved in CAD development.

**Methods:**

TBX5 gene promoter was genetically and functionally analysed in large groups of AMI patients (*n* = 432) and ethnic-matched healthy controls (*n* = 448).

**Results:**

Six novel heterozygous DNA sequence variants (DSVs) in the TBX5 gene promoter (g.4100A > G, g.4194G > A, g.4260 T > C, g.4367C > A, g.4581A > G and g.5004G > T) were found in AMI patients, but in none of controls. These DSVs significantly changed the activity of TBX5 gene promoter in cultured cells (*P* < 0.05). Furthermore, three of the DSVs (g.4100A > G, g.4260 T > C and g.4581A > G) evidently modified the binding sites of unknown transcription factors.

**Conclusions:**

The DSVs identified in AMI patients may alter TBX5 gene promoter activity and change TBX5 level, contributing to AMI development as a rare risk factor.

## Introduction

Coronary artery disease (CAD), including acute myocardial infarction (AMI), is a common complex disease. Although genome-wide association studies have identified a great number of genetic loci and variants for CAD and AMI, genetic causes and underlying mechanisms for CAD and AMI remain largely unclear [1, 2]. Recent epidemiological studies have demonstrated that incidence of CAD and AMI is strikingly higher in patients with congenital heart disease than that in normal population [3, 4]. Dysregulation of cardiac genes and embryonic developmental genes have been associated with congenital heart diseases [5–9]. Therefore, it is postulated that dysregulation of cardiac developmental genes may also contribute to the CAD development.

T-box (TBX) transcription factors are a group of factors with a highly conserved DNA-binding domain (T-box), and play critical roles in embryonic development. There are 18 T-box family members, including TBX transcriptional factor 5 [TBX5] [10]. TBX5 gene is expressed in the embryonic and adult heart. TBX5 is required in the early heart tube formation, cardiac chamber morphogenesis and cardiomyocyte differentiation. During the cardiac development, TBX5 activates multiple downstream target genes, including atrial natriuretic factor (ANF), connexin 40 (CX40) and serum response factor (SRF). TBX5 functions alone or synergistically with interacting partners, such as NK2 transcription factor related locus 5 (NKX2–5), GATA transcription factor 4 (GATA4) and TBX20 [11–15]. TBX5 is also expressed in the central conduction system, and required for the patterning and maturation of the cardiac conduction system [16, 17]. In addition, TBX5 gene is essential to limb identity and limb development [18–20]. Mutations in TBX5 gene cause Holt-Oram syndrome (HOS), which is characterized by congenital heart defects and upper limb abnormalities [21, 22]. Haploinsufficiency of TBX5 in ventricle myocytes in mice leads to diastolic dysfunction of heart, indicating that TBX5 is required for heart function in adults [23].

In human embryonic heart, TBX5 gene is expressed in myocardium, embryonic epicardium and coronary vasculature [24]. Animal experiments have shown that TBX5 regulates epicardial formation and coronary vasculogenesis in a dose-dependent manner [25]. Coronary artery abnormalities have been reported in HOS patients, further suggesting that TBX5 is involved in the coronary vessel formation [26–29]. Developmental biological studies have shown that TBX5 is a dose-sensitive regulator [30]. A gain-of-function TBX5 gene mutation is associated with atypical HOS and paroxysmal atrial fibrillation [31]. In addition, TBX5 is crucial to the cytokine gene expression in human T cells and fibroblast cells, indicating TBX5 involvement in inflammation [32, 33]. Therefore, altered levels of TBX5 may affect coronary vessel formation and inflammation, contributing to AMI development. In this study, we genetically and functionally analyzed the TBX5 gene promoter in large cohort of AMI patients and ethnic-matched healthy controls.

## Methods

### Study population

During the period from April, 2014 to August, 2016, the AMI patients (*n* = 432, male 284, female 148, median age 62.00 years) were recruited from Cardiac Care Unit, Affiliated Hospital of Jining Medical University, Jining, Shandong Province, China. AMI diagnosis criteria included typical clinical manifestations, changed ECG and biochemical markers (troponin or creatine kinase-MB), or coronary angioplasty. The ethnic-matched healthy controls (*n* = 448, male 235, female 213, median age 48.00 years) were recruited from the same hospital. The healthy controls with familial history of AMI, CAD and congenital heart diseases were excluded. This study protocol was approved by the Human Ethic Committee of Affiliated Hospital of Jining Medical University. This work was conducted according to the principles of the Declaration of Helsinki. Written informed consents were obtained from all participants.

### DNA sequencing analysis of TBX5 gene promoter

Genomic DNAs were extracted from peripheral leukocytes isolated from venous blood. DNA fragments for TBX5 gene promoter were generated by PCR and directly sequenced as previously reported [6]. PCR primers were shown in Table 1. DNA sequence variants (DSVs) were identified by comparing to wild type TBX5 gene promoter.

**Table 1:** PCR primers for the human TBX5 gene promoter

PCR primers	Sequences	Location	Position	Products
Sequencing				
TBX5-F1	5′-GGGTTTGGGAGAAGGATTTC-3′	3981	-1020 bp	688 bp
TBX5-R1	5′-GAGGCACGAGGCACTCTATT-3′	4668	-333 bp	
TBX5-F2	5′-AGAAATTGTGCCCATTGATC-3′	4593	-408 bp	677 bp
TBX5-R2	5′-TCTCCGTCTTCGCCTATCAG-3′	5269	+ 269 bp	
Functioning				
TBX5-F	5′-(KpnI)-CGCTCGGAGTTTCCCCTTTT-3′	3877	-1124 bp	1294 bp
TBX5-R	5′-(HindIII)-CGGAGGAATGAGGGTGATGAAC −3′	5170	+ 170 bp	

PCR primers are designed based on the genomic DNA sequence of the TBX5 gene

(NG_007373.1). The transcription start site is at the position of 5001 (+ 1)

### TBX5 gene promoter activity with dual-luciferase reporter assay

Wild type and variant TBX5 gene promoters (1294 bp) were generated, and inserted into pGL3-basic for reporter gene expression constructs. The constructs were transiently transfected into cultured human embryonic kidney cells (HEK-293, CRL-1573, ATCC) or rat cardiomyocyte line cells (H9c2, CRL-1446, ATCC) for 48 h. Luciferases activities of transfected cells were measured using dual-luciferase reporter assay system. Relative activity of variant TBX5 gene promoters were calculated as previously reported [6].

### Electrophoretic mobility shift assay (EMSA)

Nuclear extracts were prepared from cultured HEK-293 or H9c2 cells. As previously described, EMSA was performed with biotinylated double-stranded oligonucleotides (30 bp) to examine whether DSVs affected binding sites for transcription factors [34].

### Statistical analysis

Transfection data were expressed as means ± standard errors of the means, and analyzed using two-way analysis of variance. DSV frequencies in AMI patients and controls were compared with SPSS v23.0 (SPSS, Chicago, IL, USA). *P* < 0.05 was considered as statistically significant difference.

## Results

### Identified DSVs in TBX5 gene promoter

**Fig. 1:**
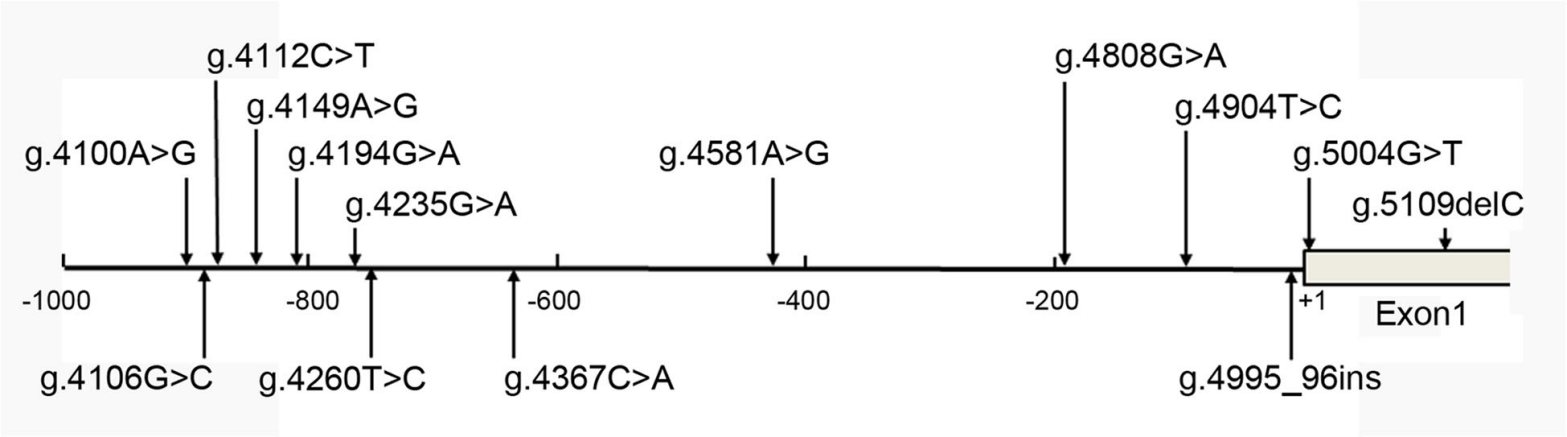
Locations of DSVs in TBX5 gene promoter. The numbers represents the genomic DNA sequences of the human TBX5 gene (GenBank accession number NG_007373.1). The transcription start site is at the position of 5001 (+ 1) in the first exon

**Fig. 2:**
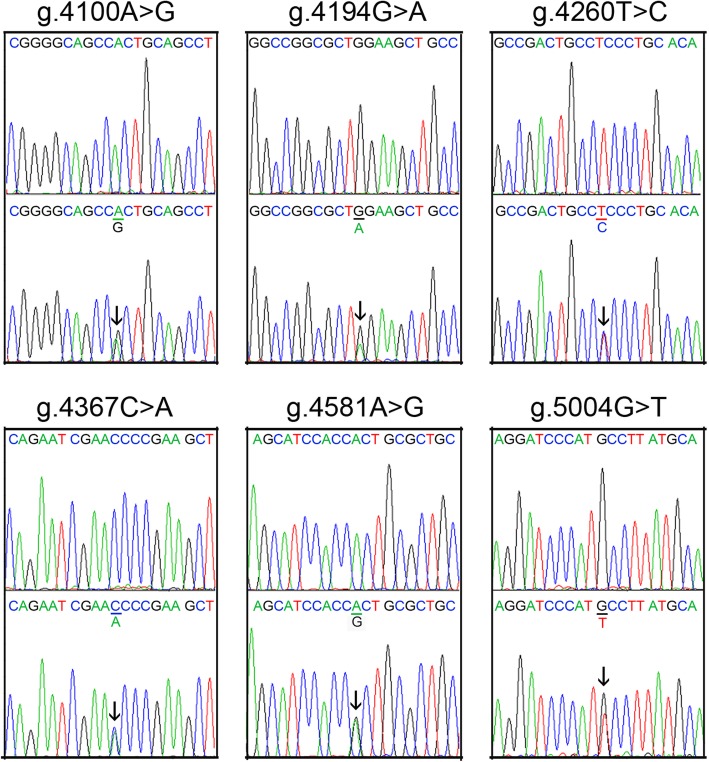
Sequencing chromatograms of the novel DSVs in the TBX5 gene promoter in AMI patients. Sequencing orientations are forward. Top panels show wild type and bottom panels heterozygous DNA sequences. Heterozygous DSVs are marked with arrows

Fourteen DSVs, including three single-nucleotide polymorphisms (SNPs), were identified in this study population (Fig. 1 and Table 2). Six novel heterozygous DSVs (g.4100A > G, g.4194G > A, g.4260 T > C, g.4367C > A, g.4581A > G and g.5004G > T) were only identified in AMI patients, the chromatograms of which were shown in Fig. 2. Four novel heterozygous DSVs (g.4149A > G, g.4904 T > C, g.4995_96insTAATAA and g.5109delC) were only found in healthy controls. Three SNPs [g.4106G > C (rs79795050), g.4112C > T (rs7957609) and g.4808G > A (rs57820630)] and one heterozygous DSV (g.4235G > A) were found in both AMI patients and controls with similar frequencies (*P* > 0.05).

**Table 2:** The DSVs within the TBX5 gene promoters in AMI patients and controls

DSVs	Genotypes	Location^1^	Controls (*n* = 448)	AMI (*n* = 432)	*P* value
g.4100A > G	AG	-901 bp	0	1	–
g.4106G > C (rs79795050)	GG	-895 bp	360	362	0.601
	GC		66	65	
	CC		2	5	
g.4112C > T (rs7957609)	CT	-889 bp	418	413	0.125
			27	19	
			3	0	
g.4149A > G	AG	-852 bp	1	0	–
g.4194G > A	GA	-807 bp	0	1	–
g.4235G > A	GA	-766 bp	3	5	0.499
g.4260 T > C	TC	-741 bp	0	1	–
g.4367C > A	CA	-634 bp	0	1	–
g.4581A > G	AG	-420 bp	0	1	–
g.4808G > A (rs57820630)	GA	-193 bp	4	5	0.748
g.4904 T > C	TC	-97 bp	2	0	–
g.4995_96insTAATAA	−/TAATAA	-6 bp	1	0	–
g.5004G > T	GT	+ 4 bp	0	1	–
g.5109delC	T/−	+ 109 bp	1	0	–

^1^, DSVs are located upstream (−) to the transcription start site of TBX5 gene at 5001 of NG_007373.1

### DSVs-affected activity of TBX5 gene promoter

**Fig. 3:**
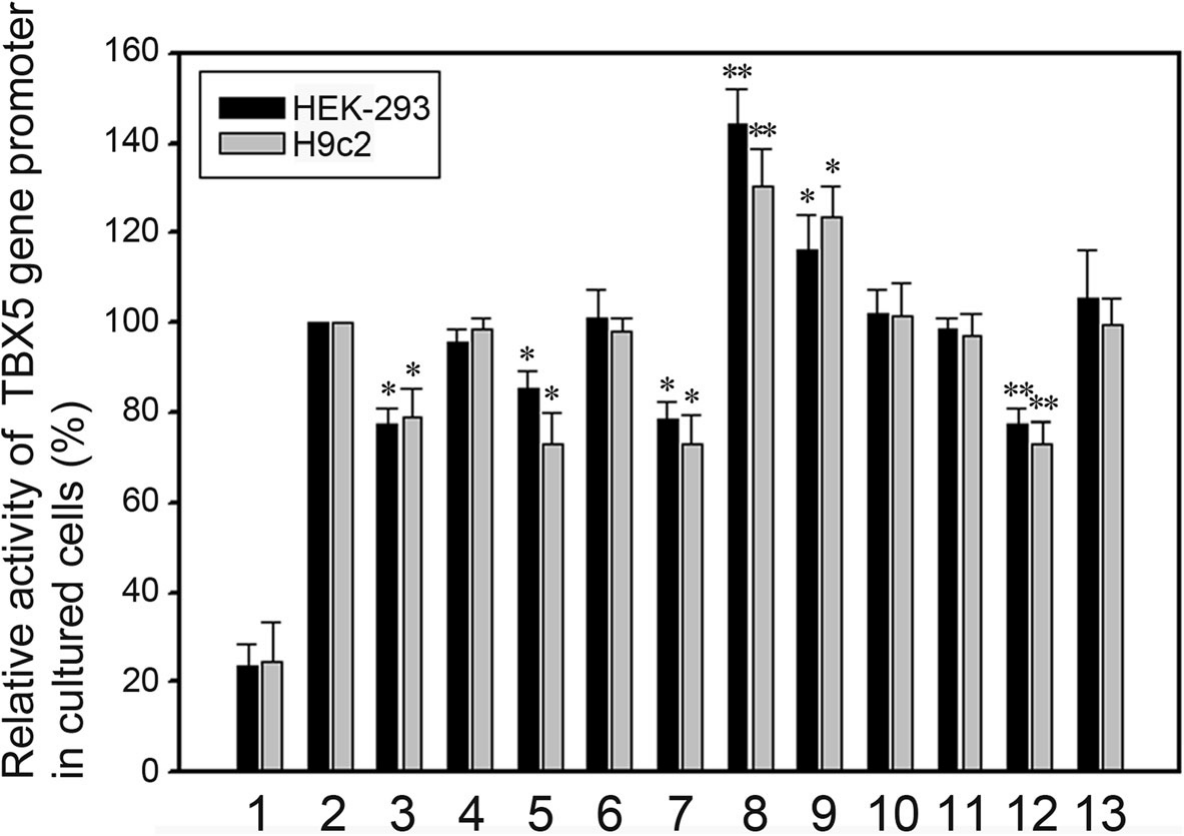
Relative activity of wild type and variant TBX5 gene promoters. Empty vector pGL3-basic was used as a negative control. Transcriptional acitivity of wild type TBX5 gene promoter was designed as 100%. Relative activities of variant TBX5 gene promoters were calculated. Lanes 1, pGL3-basic; 2, pGL3-WT; 3, pGL3-4100G; 4, pGL3-4149G; 5, pGL3-4194A; 6, pGL3-4235A; 7, pGL3-4260C; 8, pGL3-4367A; 9, pGL-4581G; 10, pGL3-4904C; 11, pGL3-4915_16insTAATAA; 12, pGL3-5004 T; and 13, pGL3-5109delC. Dark bars indicate HEK-293 cells, and grey bars H9c2 cells. WT, wild type. *, *P* < 0.05; **, *P* < 0.01

Reporter gene expression constructs, including empty pGL3-basic (negative control), pGL3-WT (wild type TBX5 gene promoter), pGL3-4100G, pGL3-4149G, pGL3-4194A, pGL3-4235A, pGL3-4260C, pGL-4367A, pGL-4581G, pGL3-4904C, pGL3-4915_16insTAATAA, pGL3-5004 T and pGL3-5109delC, were transfected into cultured HEK-293 cells. The results showed that the DSVs in AMI patients either significantly increased (g.4367C > A and g.4581A > G) or decreased (g.4100A > G, g.4194G > A, g.4260 T > C and g.5004G > T) the activity of TBX5 gene promoter (*P* < 0.05). In contrast, the DSVS (g.4149A > G, g.4904 T > C, g.4995_96insTAATAA and g.5109delC) in controls or the DSV (g.4235G > A) in both AMI patients and controls did not significantly affect activity of TBX5 gene promoter (*P* > 0.05) (Fig. 3).

To examine tissue-specific effects of the DSVs, we measured the activities of variant TBX5 gene promoter in cultured H9c2 cells. As expected, the DSV in AMI patients either significantly increased (g.4367C > A and g.4581A > G) or decreased (g.4100A > G, g.4194G > A, g.4260 T > C and g.5004G > T) the activity of TBX5 gene promoter (*P* < 0.05). Taken together, the DSVs in AMI patients significantly affect the activity of TBX5 gene promoter in a non-tissue specific manner.

### DSVs-affected putative binding sites for transcription factors

To determine whether DSVs affect putative binding sites for transcription factors, TBX5 gene promoter was analyzed with JASPAR program (http://jaspar.genereg.net/). The DSV (g.4100A > G) may modify binding sites for basic leucine zipper transcription factor nuclear respiratory factor-1 (NRF-1), nuclear factor 1 X-type (NFIX) and nuclear factor kappa B subunit 2 (NFKB2). The DSV (g.4194G > A) may abolish binding sites for E2F transcription factor 4 (E2F4), a fork head factor, and basic helix-span-helix transcription factor AP-2 Alpha (TFAP2A). The DSV (g.4260 T > C) may create a binding site for zinc finger transcriptional repressor hypermethylated in cancer 2 (HIC2) and modify binding sites for TFAP2B and TFAP2C. The DSV (g.4367C > A) may create a binding site for nuclear factor of activated T-cells 5 (NFAT5) and modify binding site for TFAP2A. The DSV (g.4581A > G) may abolish binding sites for zinc finger protein 354C (ZNF354C) and zinc finger and BTB domain-containing protein 7C (ZBTB7C). The DSV g.5004G > T may create a binding site for TEA domain transcription factor 1 (TEAD1) and abolish a binding site for transcription factor EB (TFEB). Therefore, the DSVs in AMI patients may affect putative binding sites for transcription factors.

### DSVs-affected binding site of transcription factors

**Fig. 4:**
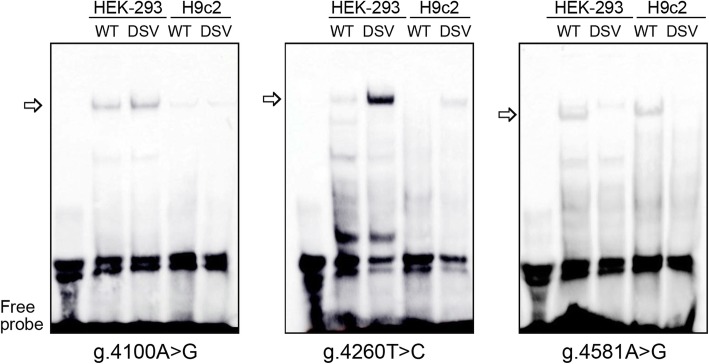
EMSA of biotin-labeled oligonucleotides. Free probe was marked with an arrow. The affected binding for a transcription factor is marked with an open arrow

To investigate whether the DSVs experimentally affect binding sites for transcription factors, EMSA was performed with wild type or variant oligonucleotides (Table 3). In both HEK293 and H9c2 cells, the DSVs (g.4100A > G and g.4260 T > C) enhanced the binding of unknown transcription factors, which were likely transcriptional repressors as both DSVs decreased the TBX5 gene promoter activity. The DSV (g.4581A > G) almost abolished the binding of an unknown transcription factor, which acted as a transcription repressor since the DSV significantly increased the TBX5 gene promoter activity (Fig. 4). Other DSVs (g.4194G > A, g.4367C > A and g.5004G > T) did not evidently affect the binding of transcription factors, likely due to EMSA sensitivity.

**Table 3:** Biotinylated double-stranded oligonucleotides for EMSA

DSVs	Oligonucleotide sequences 1	Locations
g.4100A > G	5′-AAGCTCGGGGCAGCC(**A/G**)CTGCAGCCTGGCTG-3’	4085–4114
g.4194G > A	5′-CTTGTGGCCGGCGCT(**G/A**)GAAGCTGCCCGCTC-3’	4179–4208
g.4260 T > C	5′-TGCCGGCCGACTGCC(**T/C**)CCCTGCACATTTTG-3′	4245–4274
g.4367C > A	5′-AAACCCAGAATCGAA(**C/A**)CCCGAAGCTGGGGG-3’	4352–4381
g.4581A > G	5′-TTGCGAGCATCCACC(**A/G**)CTGCGCTGCTTAGA-3’	4566–4595
g.5004G > T	5′-TAATAAGGATCCCAT(**G/T**)CCTTATGCAAGAGA-3’	4989–5018

Bold letters in the oligonuceotide sequences indicate the DSVs

## Discussion

Mutations in TBX5 gene have been implicated in HOS, a variety of congenital heart diseases, dilated cardiomyopathy and atrial fibrillation [35, 36]. Intron variants of TBX5 gene is associated with PR interval, QRS duration and QT interval, as well as atrial fibrillation [37–39]. Regulatory variation in a TBX5 gene enhancer causes isolated congenital heart disease [40]. Genetic variants and altered expression levels of TBX5 gene have been reported in patients with cancers [41, 42]. In previous studies, we have reported two heterozygous DSVs, g.4303C > G and g.4900C > T, in patients with ventricular septal defects [6]. In this study, six novel heterozygous DSVs (g.4100A > G, g.4194G > A, g.4260 T > C, g.4367C > A, g.4581A > G and g.5004G > T) were identified in six AMI patients, and significantly affected the transcriptional activity of TBX5 gene promoter. Further EMSA revealed that the DSVs modified binding sites for transcription factors. Collective frequency of the DSVs in AMI patients were 1.39% (6/432). The DSVs in TBX5 gene promoter identified in patients with ventricular septal defects were not found in this study, suggesting these DSVs were unique in AMI patients. Therefore, our findings suggested that TBX5 gene promoter DSVs may change TBX5 levels, contributing to AMI development at adult stage. As this is a single-center study, more AMI patients from multi-center will be needed to further confirm our findings.

The human TBX5 gene is localized to 12q24.1, contains 9 exons and spans more than 47 kb [21, 43]. There are GC box, T-box-like binding elements, and NKX2–5 binding site in the human TBX5 gene promoter. TBX5 gene is likely to be autoregulated at transcriptional level [44]. An enhancer located to ~ 90 kb downstream of TBX5 gene has been identified [36]. microRNAs (miR-10a and miR-10b) repress TBX5 gene expression and decrease TBX5 level by targeting the TBX5 3′-untranslated region [45]. In this study, the novel DSVs in the TBX5 gene promoter were biologically functional by affecting binding sites for transcription factors. Molecular mechanisms for these DSVs to influence TBX5 gene expression will be further explored and elucidated.

A number of downstream target genes and interacting partners for TBX5 have been reported. During the heart development, TBX5 regulates NKX2.5, atrial natriuretic factor (ANF) and cardiac structural genes [11, 46–48]. TBX5 directs SRF gene expression by binding to its 3′-untranslated region [49]. TBX5 partners include GATA4, GATA5, MEF2C, NKX2–5, and other cardiac transcription factors [11, 12, 50–53]. These transcription factors form a complex cardiac regulatory network to coordinately control cardiac gene expression [54]. TBX5 forms a positive and negative feed-forward circuit with Sal-like protein 4 (SALL4), a zinc finger transcription factor, to ensure the morphogenesis of embryonic heart [55]. A member of Krüppel-like family of zinc finger proteins, Krueppel-like factor 13 (KLF13), is co-expressed with TBX5 in cardiomyocytes, which may act as a genetic modifier of TBX5 [56]. In a gene regulatory network to maintain cardiac rhythm, TBX5 directly activates paired like homeodomain 2 (PITX2) gene [57]. In addition, TBX5 binds to the proximal region of the NFAT3 (nuclear factor of activated T cells 3) gene in human T cells [32]. Therefore, changed TBX5 level may interrupt cardiac gene regulatory network, contributing to AMI development by affecting cardiac function and influencing coronary vasculature.

## Conclusion

In this study, novel DSVs in TBX5 gene promoter were identified in AMI patients. The DSVs significantly altered TBX5 gene promoter activity in cultured cardiomyocytes. EMSA revealed that the DSVs affected binding sites for transcription factors. Therefore, TBX5 gene promoter DSVs may alter TBX5 gene promoter activity and change subsequent TBX5 level, contributing to AMI development as a rare risk factor.

## Data Availability

The datasets used and/or analyzed during the current study available from the corresponding author on reasonable request.

## Abbreviations

AMI: Acute myocardial infarction
ANF: Atrial natriuretic factor
CAD: Coronary artery disease
CX40: Connexin 40
DSVs: DNA sequence variants
E2F4: E2F transcription factor 4
GATA4: GATA transcription factor 4
HIC2: Hypermethylated in cancer 2
HOS: Holt-Oram syndrome
KLF13: Krueppel-like factor 13
NFAT3: Nuclear factor of activated T cells 3
NFAT5: Nuclear factor of activated T cells 5
NFIX: Nuclear factor 1 X-type
NFKB2: Nuclear factor kappa B subunit 2
NKX2–5: NK2 transcription factor related locus 5
NRF-1: Nuclear respiratory factor-1
PITX2: Paired like homeodomain 2
SALL4: Sal-like protein 4
SRF: Serum response factor
TBX5: T-box transcription factor 5
TEAD1: TEA domain transcription factor 1
TFAP2A: Transcription factor AP-2 alpha
TFAP2B: Transcription factor AP-2 beta
TFAP2C: Transcription factor AP-2 gamma
TFEB: Transcription factor EB
ZBTB7C: BTB domain-containing protein 7C
ZNF354C: Zinc finger protein 354C

## References

[1] Assimes TL, Roberts R (2016). Genetics: implications for prevention and Management of Coronary Artery Disease. J Am Coll Cardiol.

[2] McPherson R, Tybjaerg-Hansen A (2016). Genetics of coronary artery disease. Circ Res.

[3] Fedchenko M, Mandalenakis Z, Rosengren A, Lappas G, Eriksson P, Skoglund K (2017). Ischemic heart disease in children and young adults with congenital heart disease in Sweden. Int J Cardiol.

[4] Olsen M, Marino B, Kaltman J, Laursen H, Jakobsen L, Mahle W (2017). Myocardial infarction in adults with congenital heart disease. Am J Cardiol.

[5] Chen J, Wang S, Pang S, Cui Y, Yan B, Hawley RG (2019). Functional genetic variants of the GATA4 gene promoter in acute myocardial infarction. Mol Med Rep.

[6] Shan J, Pang S, Qiao Y, Ma L, Wang H, Xing Q (2012). Functional analysis of the novel sequence variants within TBX5 gene promoter in patients with ventricular septal defects. Transl Res.

[7] Wu G, Shan J, Pang S, Wei X, Zhang H, Yan B (2012). Genetic analysis of the promoter region of the GATA4 gene in patients with ventricular septal defects. Transl Res.

[8] Zhao JY, Yang XY, Gong XH, Gu ZY, Duan WY, Wang J (2012). Functional variant in methionine synthase reductase intron-1 significantly increases the risk of congenital heart disease in the Han Chinese population. Circulation.

[9] Zhao JY, Qiao B, Duan WY, Gong XH, Peng QQ, Jiang SS (2014). Genetic variants reducing MTR gene expression increase the risk of congenital heart disease in Han Chinese populations. Eur Heart J.

[10] Greulich F, Rudat C, Kispert A (2011). Mechanisms of T-box gene function in the developing heart. Cardiovasc Res.

[11] Bruneau BG, Nemer G, Schmitt JP, Charron F, Robitaille L, Caron S (2001). A murine model of Holt-Oram syndrome defines roles of the T-box transcription factor Tbx5 in cardiogenesis and disease. Cell.

[12] Hiroi Y, Kudoh S, Monzen K, Ikeda Y, Yazaki Y, Nagai R (2001). Tbx5 associates with Nkx2-5 and synergistically promotes cardiomyocyte differentiation. Nat Genet.

[13] Steimle JD, Moskowitz IP (2017). TBX5: a key regulator of heart development. Curr Top Dev Biol.

[14] Takeuchi JK, Ohgi M, Koshiba-Takeuchi K, Shiratori H, Sakaki I, Ogura K (2003). Tbx5 specifies the left/right ventricles and ventricular septum position during cardiogenesis. Development..

[15] Takeuchi JK, Bruneau BG (2009). Directed transdifferentiation of mouse mesoderm to heart tissue by defined factors. Nature..

[16] Moskowitz IP, Kim JB, Moore ML, Wolf CM, Peterson MA, Shendure J (2007). A molecular pathway including Id2, Tbx5, and Nkx2-5 required for cardiac conduction system development. Cell.

[17] Moskowitz IP, Pizard A, Patel VV, Bruneau BG, Kim JB, Kupershmidt S (2004). The T-box transcription factor Tbx5 is required for the patterning and maturation of the murine cardiac conduction system. Development.

[18] Rodriguez-Esteban C, Tsukui T, Yonei S, Magallon J, Tamura K, Izpisua Belmonte JC (1999). The T-box genes Tbx4 and Tbx5 regulate limb outgrowth and identity. Nature.

[19] Sheeba CJ, Logan MP (2017). The roles of T-box genes in vertebrate limb development. Curr Top Dev Biol.

[20] Takeuchi JK, Koshiba-Takeuchi K, Matsumoto K, Vogel-Höpker A, Naitoh-Matsuo M, Ogura K (1999). Tbx5 and Tbx4 genes determine the wing/leg identity of limb buds. Nature.

[21] Basson CT, Bachinsky DR, Lin RC, Levi T, Elkins JA, Soults J (1997). Mutations in human TBX5 [corrected] cause limb and cardiac malformation in Holt-Oram syndrome. Nat Genet.

[22] Li QY, Newbury-Ecob RA, Terrett JA, Wilson DI, Curtis AR, Yi CH (1997). Holt-Oram syndrome is caused by mutations in TBX5, a member of the Brachyury (T) gene family. Nat Genet.

[23] Zhu Y, Gramolini AO, Walsh MA, Zhou YQ, Slorach C, Friedberg MK (2008). Tbx5-dependent pathway regulating diastolic function in congenital heart disease. Proc Natl Acad Sci U S A.

[24] Hatcher CJ, Diman NY, Kim MS, Pennisi D, Song Y, Goldstein MM (2004). A role for Tbx5 in proepicardial cell migration during cardiogenesis. Physiol Genomics.

[25] Diman NY, Brooks G, Kruithof BP, Elemento O, Seidman JG, Seidman CE (2014). Tbx5 is required for avian and mammalian epicardial formation and coronary vasculogenesis. Circ Res.

[26] Aung TT, Roberto ES, Wase A (2016). Absent left Main coronary artery and separate Ostia of left coronary system in a patient with Holt-Oram syndrome and sinus node dysfunction. Am J Case Rep.

[27] Hurst JA, Hall CM, Baraitser M (1991). The Holt-Oram syndrome. J Med Genet.

[28] Smith AT, Sack GH, Taylor GJ (1979). Holt-Oram syndrome. J Pediatr.

[29] Vianna CB, Miura N, Pereira AC, Jatene MB (2011). Holt-Oram syndrome: novel TBX5 mutation and associated anomalous right coronary artery. Cardiol Young.

[30] Mori AD, Zhu Y, Vahora I, Nieman B, Koshiba-Takeuchi K, Davidson L (2006). Tbx5-dependent rheostatic control of cardiac gene expression and morphogenesis. Dev Biol.

[31] Postma AV, van de Meerakker JB, Mathijssen IB, Barnett P, Christoffels VM, Ilgun A (2008). A gain-of-function TBX5 mutation is associated with atypical Holt-Oram syndrome and paroxysmal atrial fibrillation. Circ Res.

[32] Kaminuma O, Kitamura N, Nishito Y, Nemoto S, Tatsumi H, Mori A (2018). Downregulation of NFAT3 due to lack of T-box transcription factor TBX5 is crucial for cytokine expression in T cells. J Immunol.

[33] Karouzakis E, Trenkmann M, Gay RE, Michel BA, Gay S, Neidhart M (2014). Epigenome analysis reveals TBX5 as a novel transcription factor involved in the activation of rheumatoid arthritis synovial fibroblasts. J Immunol.

[34] Gao F, Su Q, Yang W, Pang S, Wang S, Cui Y (2018). Functional variants in the LC3B gene promoter in acute myocardial infarction. J Cell Biochem.

[35] Su W, Zhu P, Wang R, Wu Q, Wang M, Zhang X (2017). Congenital heart diseases and their association with the variant distribution features on susceptibility genes. Clin Genet.

[36] Zhu T, Qiao L, Wang Q, Mi R, Chen J, Lu Y (2017). T-box family of transcription factor-TBX5, insights in development and disease. Am J Transl Res.

[37] Holm H, Gudbjartsson DF, Arnar DO, Thorleifsson G, Thorgeirsson G, Stefansdottir H (2010). Several common variants modulate heart rate, PR interval and QRS duration. Nat Genet.

[38] Sinner MF, Tucker NR, Lunetta KL, Ozaki K, Smith JG, Trompet S (2014). Integrating genetic, transcriptional, and functional analyses to identify 5 novel genes for atrial fibrillation. Circulation.

[39] Zhang R, Tian X, Gao L, Li H, Yin X, Dong Y (2016). Common variants in the TBX5 gene associated with atrial fibrillation in a Chinese Han population. PLoS One.

[40] Smemo S, Campos LC, Moskowitz IP, Krieger JE, Pereira AC, Nobrega MA (2012). Regulatory variation in a TBX5 enhancer leads to isolated congenital heart disease. Hum Mol Genet.

[41] Becker J, May A, Gerges C, Anders M, Schmidt C, Veits L (2016). The Barrett-associated variants at GDF7 and TBX5 also increase esophageal adenocarcinoma risk. Cancer Med.

[42] Yu J, Ma X, Cheung KF, Li X, Tian L, Wang S (2010). Epigenetic inactivation of T-box transcription factor 5, a novel tumor suppressor gene, is associated with colon cancer. Oncogene.

[43] Yi CH, Russ A, Brook JD (2000). Virtual cloning and physical mapping of a human T-box gene, TBX4. Genomics.

[44] Sun G, Lewis LE, Huang X, Nguyen Q, Price C, Huang T (2004). TBX5, a gene mutated in Holt-Oram syndrome, is regulated through a GC box and T-box binding elements (TBEs). J Cell Biochem.

[45] Wang F, Yang XY, Zhao JY, Yu LW, Zhang P, Duan WY (2014). miR-10a and miR-10b target the 3′-untranslated region of TBX5 to repress its expression. Pediatr Cardiol.

[46] Ghosh TK, Packham EA, Bonser AJ, Robinson TE, Cross SJ, Brook JD (2001). Characterization of the TBX5 binding site and analysis of mutations that cause Holt-Oram syndrome. Hum Mol Genet.

[47] Linhares VL, Almeida NA, Menezes DC, Elliott DA, Lai D, Beyer EC (2004). Transcriptional regulation of the murine Connexin40 promoter by cardiac factors Nkx2-5, GATA4 and Tbx5. Cardiovasc Res.

[48] Plageman TF, Yutzey KE (2006). Microarray analysis of Tbx5-induced genes expressed in the developing heart. Dev Dyn.

[49] Barron MR, Belaguli NS, Zhang SX, Trinh M, Iyer D, Merlo X (2005). Serum response factor, an enriched cardiac mesoderm obligatory factor, is a downstream gene target for Tbx genes. J Biol Chem.

[50] Garg V, Kathiriya IS, Barnes R, Schluterman MK, King IN, Butler CA (2003). GATA4 mutations cause human congenital heart defects and reveal an interaction with TBX5. Nature.

[51] Ghosh TK, Song FF, Packham EA, Buxton S, Robinson TE, Ronksley J (2009). Physical interaction between TBX5 and MEF2C is required for early heart development. Mol Cell Biol.

[52] Maitra M, Schluterman MK, Nichols HA, Richardson JA, Lo CW, Srivastava D (2009). Interaction of Gata4 and Gata6 with Tbx5 is critical for normal cardiac development. Dev Biol.

[53] Misra C, Chang SW, Basu M, Huang N, Garg V (2014). Disruption of myocardial Gata4 and Tbx5 results in defects in cardiomyocyte proliferation and atrioventricular septation. Hum Mol Genet.

[54] Luna-Zurita L, Stirnimann CU, Glatt S, Kaynak BL, Thomas S, Baudin F (2016). Complex interdependence regulates heterotypic transcription factor distribution and coordinates Cardiogenesis. Cell.

[55] Koshiba-Takeuchi K, Takeuchi JK, Arruda EP, Kathiriya IS, Mo R, Hui CC (2006). Cooperative and antagonistic interactions between Sall4 and Tbx5 pattern the mouse limb and heart. Nat Genet.

[56] Darwich R, Li W, Yamak A, Komati H, Andelfinger G, Sun K (2017). KLF13 is a genetic modifier of the Holt-Oram syndrome gene TBX5. Hum Mol Genet.

[57] Nadadur RD, Broman MT, Boukens B, Mazurek SR, Yang X, van den Boogaard M (2016). Pitx2 modulates a Tbx5-dependent gene regulatory network to maintain atrial rhythm. Sci Transl Med.

